# RpoS integrates CRP, Fis, and PhoP signaling pathways to control *Salmonella* Typhi *hlyE* expression

**DOI:** 10.1186/1471-2180-14-139

**Published:** 2014-05-31

**Authors:** Matías R Jofré, Leonardo M Rodríguez, Nicolás A Villagra, Alejandro A Hidalgo, Guido C Mora, Juan A Fuentes

**Affiliations:** 1 Facultad de Ciencias Biológicas, Universidad Andres Bello, Santiago, Chile; 2 Facultad de Medicina, Universidad Andres Bello, Santiago, Chile; 3 Laboratorio de Microbiología, Universidad Andres Bello, República 217, 2° Piso, Santiago, Chile

**Keywords:** *hlyE*, *rpoS*, *phoP*, cAMP-receptor protein, Fis protein, Catabolite repression

## Abstract

**Background:**

SPI-18 is a pathogenicity island found in some *Salmonella enterica* serovars, including *S.* Typhi. SPI-18 harbors two ORFs organized into an operon, *hlyE* and *taiA* genes, both implicated in virulence. Regarding the *hlyE* regulation in *S.* Typhi, it has been reported that RpoS participates as transcriptional up-regulator under low pH and high osmolarity. In addition, CRP down-regulates *hlyE* expression during exponential growth. Previously, it has been suggested that there is another factor related to catabolite repression, different from CRP, involved in the down-regulation of *hlyE*. Moreover, PhoP-dependent *hlyE* up-regulation has been reported in bacteria cultured simultaneously under low pH and low concentration of Mg^2+^. Nevertheless, the relative contribution of each environmental signal is not completely clear. In this work we aimed to better understand the regulation of *hlyE* in *S.* Typhi and the integration of different environmental signals through global regulators.

**Results:**

We found that Fis participates as a CRP-independent glucose-dependent down-regulator of *hlyE*. Also, Fis and CRP seem to exert the repression over *hlyE* through down-regulating *rpoS*. Moreover, PhoP up-regulates *hlyE* expression via *rpoS* under low pH and low Mg^2+^ conditions.

**Conclusions:**

All these results together show that, at least under the tested conditions, RpoS is the central regulator in the *hlyE* regulatory network, integrating multiple environmental signals and global regulators.

## Background

The genus *Salmonella* includes two species, *Salmonella bongori* and *Salmonella enterica. S. enterica* is comprised of six subspecies where only a small fraction of the subspecies I serovars are involved in infections of humans and other warm-blooded animals [1,2]. Most of these *Salmonella* serovars are “generalists”, infecting a wide range of hosts and causing different symptoms. This is the case for *S. enterica* serovar Typhimurium (*S*. Typhimurium) and *S. enterica* serovar Enteritidis (*S.* Enteritidis) [3]. In contrast, some serovars are able to infect a specific host, causing typhoid-like disease [4]. These include *S. enterica* serovar Typhi (*S.* Typhi), which infects only humans.

*S. enterica* infection begins with ingestion of contaminated water or food. Some environmental conditions found in the gut induce the expression of virulence factors that participate in the intestinal invasion and colonization, including genes found in the *Salmonella* Pathogenicity Island 1 (SPI-1) [5]. Among these conditions, high osmolarity, microaerobiosis and response to bile seem to be the most important signals at this stage of the infection [5,6]. Some *S. enterica* serovars, such as *S.* Typhi in humans, can enter the host bloodstream, disseminate and survive inside the macrophages by expressing a different subset of genes, including SPI-2 genes [7]. The most important conditions found at this stage of the infection include nutrient depletion (especially Mg^2+^) and low pH [8]. It is thought that *Salmonella* virulence factors are specifically expressed at determined stages of the infection. Nevertheless, at present it is more obvious that several virulence factors are not restricted to a unique stage of the infection. For example, SipA, encoded by SPI-1 enhances entry efficiency to intestinal epithelial cells by promoting actin polymerization, but also contributes to proliferation of *Salmonella* inside macrophages [9].

The SPI-18 is a pathogenicity island found in a subset of *S. enterica* serovars able to produce a systemic disease in humans, including *S.* Typhi; but absent in generalist serovars such as *S.* Typhimurium [10]. The SPI-18 harbors only two ORFs organized into an operon, the *taiA – hlyE* genes [10,11]. TaiA is a novel invasin involved in increased phagocytosis of the bacteria by macrophages [11]. On the other hand, HlyE (also called ClyA or SheA) is a well characterized hemolysin that is exported in outer membrane vesicles [12]. Human infections by *S.* Typhi cause a specific antibody response to HlyE, indicating effective toxin production during the normal infective cycle [13]. Moreover, heterologous expression of *S.* Typhi *hlyE* in *S.* Typhimurium improves the colonization of deep organs in mice [10].

Regarding *hlyE* regulation in *S.* Typhi, previously it has been reported that RpoS participates as a transcriptional positive regulator under low pH and high osmolarity [14]. In addition, the global regulator CRP, implicated in catabolite repression, down-regulates *hlyE* expression during exponential growth. Nevertheless, addition of glucose to the growth medium results in a decrease of *hlyE* mRNA in *S.* Typhi Δ*crp* mutant, suggesting that there is another factor related to catabolite repression, different from CRP, involved in down-regulation of *hlyE* in *S.* Typhi [14]. PhoP-dependent up-regulation of *hlyE* was reported when bacteria were cultured simultaneously under low pH and low concentration of Mg^2+^[11]. Nevertheless, the relative contribution of each environmental signal is not clear.

In this work we wanted to better understand the regulation of *hlyE* in *S.* Typhi and the integration of different environmental signals through global regulators. We found that Fis participates as a CRP-independent glucose-dependent down-regulator of *hlyE* in *S*. Typhi. Moreover, Fis and CRP seem to exert the repression over *hlyE* via down-regulating *rpoS*. On the other hand, PhoP up-regulates *hlyE* expression via *rpoS* under low pH and low Mg^2+^ conditions. All these results together show that, at least under the tested conditions, RpoS is the central regulator in the *hlyE* regulatory network, integrating multiple environmental signals.

## Results

### Fis participates in the repression of *hlyE* at transcriptional level

**Figure 1:**
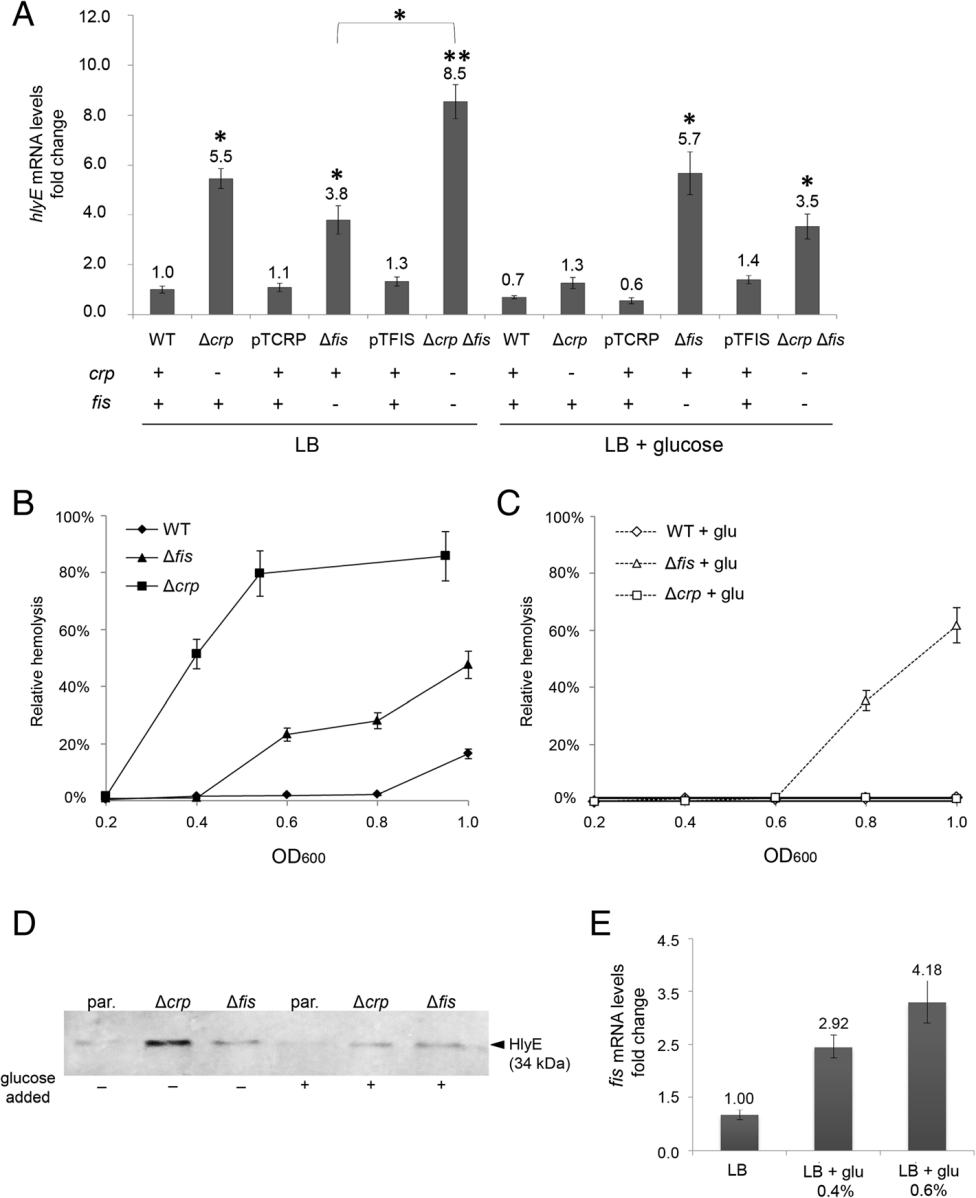
**Fis participates in down-regulation of *hlyE *expression in *S. *Typhi. A)** RT-PCR assay for mRNAs examining transcription of the *hlyE* gene from *S.* Typhi wild-type (STH2370), Δ*crp*, Δ*fis*, Δ*crp* Δ*fis* double mutant, and the complemented strains Δ*crp*/pTCRP and Δ*fis*/pTFIS. All the strains were cultured as described in Methods. *p < 0.05; **p < 0.01 (ANOVA). **B)** Relative hemolysis, obtained from the *S.* Typhi STH2370 wild type, Δ*fis* and Δ*crp* mutants. All the strains were grown in LB and samples were taken to perform the hemolytic activity quantification (see Methods). The total hemolytic activity was calculated deducting relative hemolysis of *S.* Typhi Δ*hlyE*[10] from *S.* Typhi wild type, deducting relative hemolysis of *S.* Typhi Δ*hlyE* Δ*fis* from *S.* Typhi Δ*fis* or *S.* Typhi Δ*hlyE* Δ*crp* from *S.* Typhi Δ*crp,* respectively. **C)** Relative hemolysis, obtained from the *S.* Typhi STH2370 wild type, Δ*fis* and Δ*crp* mutants grown in LB + 0.4% glucose. Hemolysis was calculated as described in B. **D)** Immunodetection of epitope-tagged HlyE from *S.* Typhi *hlyE*-3xFLAG (par.: parental strain), *S.* Typhi *hlyE*-3xFLAG Δ*crp*, and *S.* Typhi *hlyE*-3xFLAG Δ*fis*. Bacteria were cultured as described in Methods. **E)** RT-PCR for *fis* transcript levels detection in *S*. Typhi wild type strain grown to logarithmic phase in LB or LB + glucose. In all the cases, the data correspond to mean values of three independent experiments performed in triplicate. Error bars correspond to the SD.

CRP is a regulator that acts as a cAMP receptor. The cAMP-CRP complex is activated when glucose is scarce in the culture medium, consequently, numerous genes are up-/down-regulated [15]. CRP is a transcriptional regulator previously reported as an activator of the *hlyE* transcription in *E. coli*[16]. Nevertheless, genetic experiments showed that CRP participates in the negative regulation of *hlyE* in *S.* Typhi since *crp* deletion led to increased β-galactosidase activity associated to the Δ*hlyE*::*lacZ* strain [14]. Moreover, *hlyE* presented a CRP-independent down-regulation when glucose is present, suggesting the presence of another down-regulator [14]. Fis (factor for inversion simulation) is a nucleoid-associated protein that participates in the structuring of the bacterial chromosome, also known to interact at specific promoters to regulate gene transcription in *E. coli* and *S. enterica*[17-19]. Since Fis has been implicated in catabolite repression in *E. coli*[20,21], we assessed the role of Fis in *hlyE* transcription in *S.* Typhi by RT-PCR. As shown in Figure 1A, the Δ*fis* mutant presented increased transcription of *hlyE* compared with the WT, similar to the levels detected in the Δ*crp* mutant (control). In presence of glucose, the increased transcriptional level of *hlyE* seen in the Δ*crp* mutant was reverted, consistently with the presence of additional unknown glucose-dependent down-regulation [14]. On the other hand, the presence of glucose was unable to restore the normal transcriptional levels of *hlyE* in the Δ*fis* mutant (Figure 1A), showing that Fis is the CRP-independent down-regulator factor previously stated by Fuentes et al. [14], at least under laboratory conditions. In all cases, the complementation with the corresponding gene cloned into a plasmid reverted the mutant phenotype with respect to *hlyE* transcription (Figure 1A). Moreover, we found no epistasis between *crp* and *fis* with respect to *hlyE* transcription in LB with or without glucose added (Figure 1A). Further, the *crp* and *fis* mutations seemed to produce additive effects on *hlyE* transcription, strongly suggesting that these two repressors act through different pathways to repress *hlyE* expression.

To corroborate whether an increase in the *hlyE* transcript due to the absence of *fis* in LB and in LB + glucose resulted in increased HlyE-associated hemolytic activity, erythrocyte damage produced by whole bacterial lysates of *S.* Typhi WT and Δ*fis* mutants grown in LB or LB + glucose was compared by quantifying the release of hemoglobin. We used PBS and 5% sodium deoxycholate to obtain the value for 0% and 100% hemolysis respectively, as previously described [14]. As shown in Figure 1B and C, the Δ*crp* mutant appeared to be more hemolytic that the WT strain only when cultured in LB (control). As previously reported, this effect is abolished in presence of glucose [14]. On the other hand, the Δ*fis* mutant appeared also to be more hemolytic than the WT strain. However, unlike the effect observed with the Δ*crp* mutant, the presence of glucose was unable to revert the hemolysis to the WT level in the Δ*fis* strain, consistently with the results obtained with RT-PCR (Figure 1A). *S.* Typhi Δ*crp* Δ*hlyE* and *S.* Typhi Δ*fis* Δ*hlyE* exhibited no hemolysis in any condition (data not shown). To corroborate the data obtained by RT-PCR and by the determination of the hemolytic activity, we constructed the *S.* Typhi *hlyE*-3xFLAG strain by placing a 3xFLAG at the C-terminus of *hlyE*. This procedure led to subsequent detection of the FLAG-tagged proteins via Western blotting as previously described [22]. Then, we constructed the *S.* Typhi *hlyE*-3xFLAG Δ*crp* and *S.* Typhi *hlyE*-3xFLAG Δ*fis* derivatives by electrotransforming with gDNA from single mutants as described [23]. These strains were used to determine the HlyE accumulation. As shown in Figure 1D, all the results obtained by RT-PCR were confirmed by Western blot. These results together show that *fis* participates in the repression of *hlyE* at transcriptional level.

### Glucose increases transcription of *fis*

As stated above, the effect of Δ*crp* mutant on *hlyE* transcriptional level is relieved when glucose is added into the culture medium, suggesting that a second repressor is activated in some way under a glucose-rich medium. Considering that Fis also participates as an *hlyE* repressor and considering that glucose is unable to relief the Δ*fis* effect in the *hlyE* transcription (Figure 1A), we inferred that glucose might increase the transcription of *fis*. In order to test this hypothesis, we performed a RT-PCR to detect the *fis* transcript of *S.* Typhi cultured to OD_600_ = 0.4 in LB or LB + glucose. As shown in Figure 1E, the presence of glucose increased *fis* transcription compared with bacteria grown in standard LB. This result supports the fact that glucose increases the *fis* expression, thereby contributing to the repression of *hlyE*.

### CRP and Fis participate in the repression of *hlyE* transcription by repressing *rpoS* transcription

**Figure 2:**
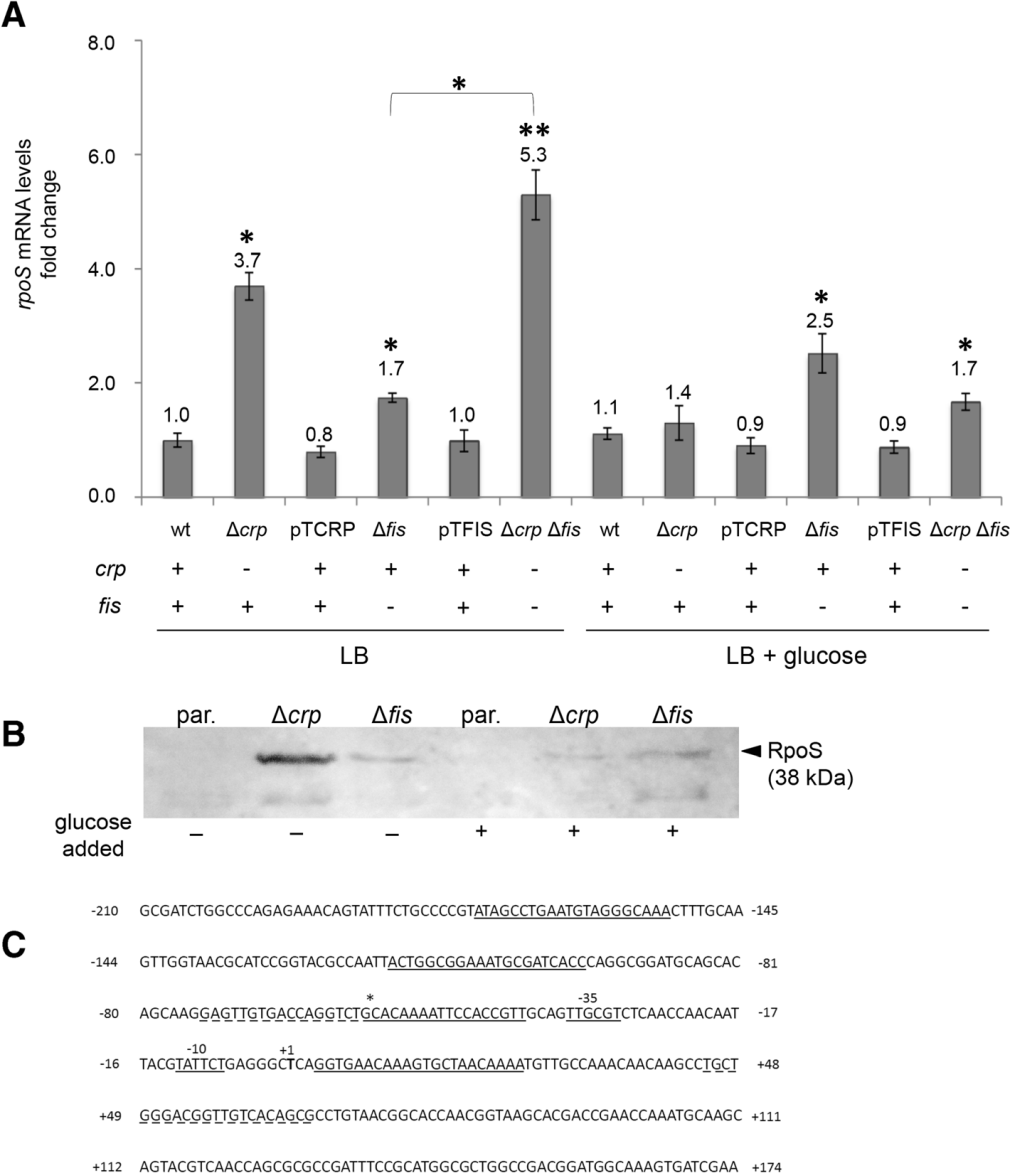
**CRP and Fis participate in the repression of *rpoS*. A)** RT-PCR assay for mRNAs examining transcription of the *rpoS* gene from *S.* Typhi wild-type (STH2370), Δ*crp*, Δ*fis*, Δ*crp* Δ*fis* double mutant, and the complemented strains Δ*crp*/pTCRP and Δ*fis*/pTFIS. All the strains were grown in LB buffered at pH 7.0 to an OD_600_ = 0.35, washed three times with PBS prior to be resuspended with LB (control) or LB + 0.4% glucose, and incubated 1 h at 37°C. After the incubation, total RNA were isolated for each strain. The same amount of RNA was applied for RT-PCR. The transcription levels were normalized against the 16 s transcript in all cases. The data correspond to mean values of three independent experiments performed in triplicate. Error bars correspond to the SD. *p < 0.05; **p < 0.001 (ANOVA). **B)** Immunodetection of epitope-tagged RpoS from *S.* Typhi *hlyE*-3xFLAG (par.: parental strain), *S.* Typhi *rpoS*-3xFLAG Δ*crp*, and *S.* Typhi *hlyE*-3xFLAG Δ*fis*. Bacteria were grown in LB buffered at pH 7.0 to an OD_600_ = 0.35, washed three times with PBS prior to be resuspended with LB or LB + 0.4% glucose, and incubated 1 h at 37°C. After the incubation, total proteins were obtained, and 20 μg were used to detect RpoS accumulation as described in Methods. **C)***In silico* sequence analysis of CRP and Fis binding boxes in *S*. Typhi CT18 *rpoS* promoter. The *S*. Typhi *rpoS* promoter contains conserved binding boxes of *S*. Typhimurium *rpoS* promoter according to the previous evidence [19,25]. Segmented underlined sequences indicate putative CRP boxes. Underlined sequences indicate putative Fis boxes. Asterisk shows a shared region of each box at the marked position. In bold, transcription start site, and short underlined sequences indicate predicted −35 and −10 promoter regions.

To find out whether CRP is repressing *hlyE* expression through a direct binding to the respective promoter region, we performed a shift mobility assay using purified CRP activated with cAMP and the DNA corresponding to the *taiA* – *hlyE* promoter region. Nevertheless, we were unable to find direct binding (data not shown). This result suggested that CRP is participating in *hlyE* repression through an indirect pathway. Previously, it has been reported that *rpoS* expression is repressed by the cAMP-CRP complex in *Vibrio vulnificus*[24]. In *Salmonella*, previously reported contradictory data indicate that CRP is an *rpoS* activator or an *rpoS* repressor [19,25]. RpoS seems to be one of the most important factors participating in *hlyE* expression, since the detection of these transcripts is not possible in Δ*rpoS* mutants when grown in pH 5.0, high osmolarity or even in standard LB [14]. Thus, CRP might repress *hlyE* expression by repressing *rpoS* in *S.* Typhi. To determine the role of CRP in *rpoS* expression, we assessed *rpoS* expression by RT-PCR. As shown in Figure 2A, the *rpoS* transcription increased in the Δ*crp* mutant compared with the WT strain. Nevertheless, the *rpoS* transcriptional levels obtained from the Δ*crp* mutant were comparable to those of the WT strain in presence of glucose, following the same pattern for *hlyE* transcript in Figure 1A. This result suggests that *crp* participates as a transcriptional repressor of *rpoS* in *S.* Typhi. Since *rpoS* is regulated at multiple levels [26], we determined whether the changes in the *rpoS* transcription also lead to changes in RpoS accumulation. For that, we performed a Western blot comparing RpoS accumulation in the *S.* Typhi *rpoS*-3xFLAG and *S.* Typhi *rpoS*-3xFLAG Δ*crp* strains. As shown in Figure 2B, RpoS from *S.* Typhi *rpoS*-3xFLAG was practically undetectable under the tested conditions, consistently with the fact that the RpoS accumulation is prevented at logarithmic phase [27]. On the other hand, the lack of *crp* led to an evident RpoS accumulation. Similarly to the data observed for *hlyE* expression (Figure 1), these results suggest that *crp* is an *rpoS* repressor and, in presence of glucose, there is another *crp*-independent repressor. The obvious candidate to be such a repressor is the transcriptional regulator Fis. Then, we assessed the role of *fis* in the *rpoS* transcription and RpoS accumulation as previously described for *crp*. As shown in Figure 2A, the Δ*fis* mutant presented a slight increase in the *rpoS* transcriptional level compared with the WT strain. Again, the addition of glucose was unable to abolish the increase of *rpoS* transcript in the Δ*fis* mutant (Figure 2A). Moreover, the lack of *fis* clearly contributed to the RpoS accumulation even in presence of glucose, as inferred from Figure 2B. All these results are consistent with the presence of CRP and Fis DNA-binding boxes at the *rpoS* promoter region in *S.* Typhimurium [19,25], which are also conserved in *S*. Typhi *rpoS* promoter (Figure 2C). Altogether, these results suggest that CRP and Fis are able to repress the *rpoS* expression by direct binding, thereby regulating *hlyE* expression in *S.* Typhi.

### PhoPQ two-component regulatory system participates in *hlyE* induction via *rpoS*

**Figure 3:**
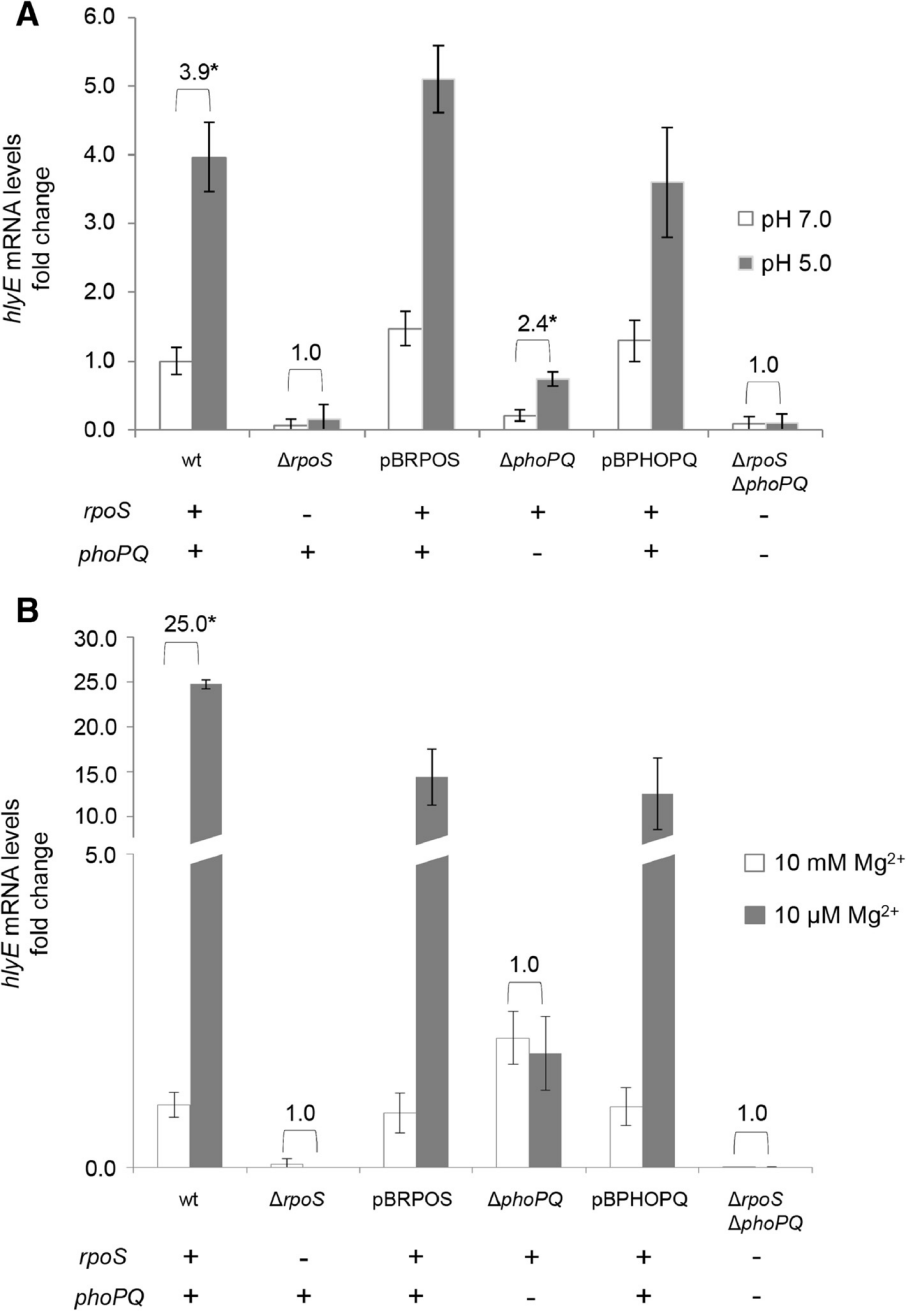
**PhoPQ two-component regulatory system participates in the *hlyE *induction via *rpoS. *A)** RT-PCR assay for mRNAs examining transcription of the *hlyE* gene from *S.* Typhi wild-type (STH2370), Δ*rpoS*, Δ*phoPQ*, and the Δ*rpoS* Δ*phoPQ* double mutant, and the complemented strains Δ*rpoS*/pBRPOS and Δ*phoPQ*/pBPHOPQ. All the strains were grown in LB buffered at pH 7.0 to an OD_600_ = 0.35, washed three times with PBS prior to be resuspended with LB buffered at pH 7.0 (control) or LB buffered at pH 5.0, and incubated 1 h at 37°C. After the incubation, total RNA were isolated for each strain. The same amount of RNA was applied for RT-PCR. The transcription levels were normalized against the 16 s transcript in all cases. The data correspond to mean values of three independent experiments performed in triplicate. Error bars correspond to the SD. **B)** RT-PCR assay for mRNAs examining transcription of the *hlyE* gene from *S.* Typhi wild-type (STH2370), Δ*rpoS*, Δ*phoPQ*, and the Δ*rpoS* Δ*phoPQ* double mutant. All the strains were grown in LB buffered at pH 7.0 to an OD_600_ = 0.35, washed three times with PBS prior to be resuspended with M9 10 mM Mg^2+^(control) or M9 10 μM Mg^2+^, and incubated 1 h at 37°C. After the incubation, total RNA were isolated for each strain. The same amount of RNA was applied for RT-PCR. The transcription levels were normalized against the 16 s transcript in all cases. The data correspond to mean values of three independent experiments performed in triplicate. Error bars correspond to the SD. In all the cases, the numbers above the bars represent the fold of induction. *p < 0.05 (ANOVA).

As shown above, we identified two genes, *crp* and *fis*, implicated in the down-regulation of *hlyE*. In addition, it has been described two global regulators implicated in the up-regulation of this gene: RpoS and PhoP. RpoS-dependent transcriptional up-regulation in low pH and high osmolarity was reported [14]. PhoP-dependent up-regulation was observed when bacteria were cultured simultaneously under low pH and low concentration of Mg^2+^ (10 μM MgCl_2_) [11]. Nevertheless, considering that the low pH is an environmental signal that activates both the RpoS- and PhoP-dependent up-regulation of *hlyE*, it is not clear the relative contribution of each global regulator to the up-regulation of this gene. Moreover, the relative contribution of low pH and low Mg^2+^ concentrations to the PhoP-dependent up-regulation is also unknown, since, as stated, these two conditions were tested at the same time [11]. In order to better understand the up-regulation of *hlyE*, we compared the transcription induction of this gene in LB pH 5.0 versus LB pH 7.0, and in minimal medium 9 (M9) 10 μM Mg^2+^ versus M9 10 mM Mg^2+^ by RT-PCR in different genetic backgrounds. As shown in Figure 3A, pH 5.0 is an inducer condition of the *hlyE* expression, as previously reported [14]. Nevertheless, the relative expression of *hlyE* in the Δ*rpoS* mutant is nearly zero, even under pH 5.0. This result suggests that *rpoS* is necessary for the induction of *hlyE* expression under low pH, but also to maintain a basal expression of *hlyE*. This result strongly suggests that *phoPQ* is unable to induce the expression of *hlyE* under low pH when *rpoS* is absent. The induction levels obtained from the Δ*rpoS* Δ*phoPQ* double mutant reinforces this hypothesis, highlighting the fact that *rpoS* is epistatic over *phoPQ*. On the other hand, the Δ*phoPQ* mutant shows the half of induction under low pH compared with the WT strain, suggesting that *rpoS*, by itself, is sufficient to exert a partial induction under low pH. Thus, both *rpoS* and *phoPQ* are necessary to achieve a complete induction under low pH of *hlyE* in *S.* Typhi. However, the *phoPQ*-dependent up-regulation occurs via RpoS. The Figure 3B shows that 10 μM Mg^2+^ is also an inducer condition, exhibiting approximately 25 fold compared with 10 mM Mg^2+^. Again, the relative expression of *hlyE* in the Δ*rpoS* mutant is nearly zero under high or low Mg^2+^ concentration, supporting the fact that *rpoS* is essential for the *hlyE* expression under different culture conditions. In addition, the induction under 10 μM Mg^2+^ is completely abolished in the Δ*phoPQ* mutant, strongly suggesting that *phoPQ* is essential to increase the *hlyE* transcript under low Mg^2+^. Moreover, *rpoS* alone is not sufficient for increase the *hlyE* transcript under the same tested conditions. Finally, *rpoS* is epistatic over *phoPQ*, reinforcing the fact that the induction of the *hlyE* expression under low concentration of Mg^2+^ depends on PhoPQ and occurs via RpoS. In all the cases, the induction was restored when the mutants were complemented with the corresponding genes cloned into a plasmid vector (Figure 3).

All these results together strongly suggest that RpoS is essential for *hlyE* transcription under all the conditions tested. On the other hand, *phoPQ* participates in *hlyE* induction under pH 5.0 and low Mg^2+^ in an RpoS-dependent manner.

## Discussion

**Figure 4:**
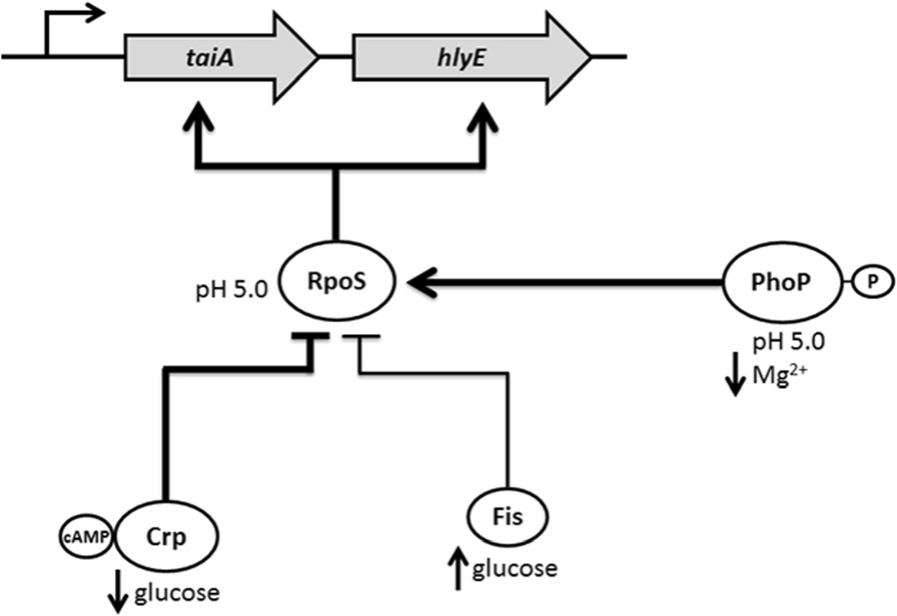
**Model showing that CRP, Fis and PhoPQ regulate *taiA-hlyE *operon expression via RpoS.** RpoS integrates multiple environmental signals through different global regulators in order to regulate the *taiA-hlyE* operon expression. Like *hlyE*, *taiA* expression is up-regulated by RpoS and PhoPQ under low pH and low Mg^2+^ concentrations [11,14].

In this work we studied the regulation of *hlyE* expression and the integration of different environmental signals through global regulators (Figure 4).

As previously reported, CRP participates in the *hlyE* repression. Nevertheless, the mechanism involved in such repression has not been elucidated [14]. The absence of obvious CRP DNA-binding boxes at the promoter region of *taiA* – *hlyE* operon [14], and the fact that we were unable to find direct binding between CRP-cAMP and the *taiA* – *hlyE* promoter region, suggests that CRP might be acting indirectly, more likely regulating other transcriptional regulators. Here, we present genetic evidence indicating that CRP exerts down-regulation of *hlyE* by repressing *rpoS* (Figures 1 and 2), one of the most important *hlyE* activators in *S.* Typhi [14]. CRP has been also described as an RpoS repressor in other bacterial species. For instance, *E. coli* Δ*crp* is remarkably resistant to hydrogen peroxide and acid stress, due to an elevated catalase activity attributable to enhanced accumulation of the alternate sigma factor RpoS [28]. On the other hand, it has been reported that *rpoS* expression is repressed by direct binding of CRP-cAMP to its promoter region in *Vibrio vulnificus*[24]. In that same work, the authors suggest that CRP could repress RpoS expression in *S. enterica* by direct binding arguing that *S. enterica rpoS* promoter presents similar CRP binding boxes than *V. vulnificus*[24]. Nevertheless, the available studies of *S. enterica* include experiments performed only with *S.* Typhimurium, and the data are controversial. Cheng and Sun demonstrated direct binding of CRP to the *rpoS* promoter region in *S.* Typhimurium, where it is able to bind to two sites in this sequence. Both sites are conserved in *S*. Typhi *rpoS* promoter, suggesting a direct binding by CRP. Moreover, the authors showed that base-substitutions performed in the CRP-binding sites at the *rpoS* promoter region, led to a decreased *rpoS* transcription, concluding that CRP is an *rpoS* up-regulator [25]. Nevertheless, this result can be also explained by the fact that the base-substitution may be affecting unknown overlapped regulators’ binding sites. On the other hand, Hirsch and Elliott showed that *S.* Typhimurium *rpoS*-*lacZ* Δ*crp* exhibited twice more β-galactosidase activity compared with the otherwise isogenic strain *S.* Typhimurium *rpoS*-*lacZ*, suggesting that CRP is an RpoS repressor [19], supporting our findings in *S.* Typhi. In addition, CRP has been described as a pleiotropic factor in *S. enterica* implicated in carbon source utilization, synthesis of flagella, reduced growth rate, and attenuation of virulence, among other functions [29-32]. This versatility can be explained, at least in part, by the fact that CRP is regulating *rpoS*.

As previously stated, *hlyE* presents a CRP-independent down-regulation when glucose is present in the culture medium, suggesting the presence of another glucose-dependent down-regulator [14]. In this work, we found that *fis* participates in *hlyE* repression especially in presence of glucose (Figure 1). It has been stated that Fis regulates gene expression by increasing/decreasing its level in *E. coli*[20]. In this work, we found that glucose induces the *fis* expression in *S.* Typhi, suggesting that Fis is also regulated by its level in *S. enterica* (Figure 1E). On the other hand, Fis precludes the RpoS accumulation by repressing the *rpoS* transcription (Figure 2). Hirsch and Elliott showed that *fis* represses *rpoS* expression by direct binding in *S.* Typhimurium [19], supporting our observations. As with CRP, Fis might participate in down-regulation of *hlyE* via RpoS, and this regulation could be exerted directly due to the presence of several putative Fis binding boxes in *S*. Typhi *rpoS* promoter (Figure 2C). Considering that *crp* and *fis* deletions seem to produce additive effects in *hlyE* transcription (Figure 1A), these two repressors probably act through different pathways to repress the *hlyE* expression (Figure 4).

We also explored the relative participation of *rpoS* and *phoPQ* in the up-regulation of *hlyE* under pH 5.0 and low Mg^2+^ concentrations. We found that RpoS is essential for *hlyE* expression under all the tested conditions, strongly suggesting that RpoS accumulation is the most relevant event in *hlyE* regulation. Moreover, both *rpoS* and *phoPQ* are necessary to achieve a complete induction of *hlyE* under low pH in *S.* Typhi, where the *phoPQ*-dependent induction occurs via RpoS. The induction pathway of RpoS by low pH is not fully understood. One component of induction is RpoS stabilization, where PhoPQ participates by serving as a transcriptional activator of the *iraP* (*yaiB*) gene in *S.* Typhimurium. IraP enhances RpoS stability by interacting with RssB, the protein that normally delivers RpoS to the ClpXP protease for degradation [33], supporting the fact that *phoPQ* requires *rpoS* to induce *hlyE* expression (Figure 4). On the other hand, it has been reported that mutant strains unable to degrade RpoS still exhibited induction under acid shock in *S. enterica*. Moreover, *rpoS* translation is enhanced under low pH [34]. This phenomenon can explain the fact that *rpoS* is able to partially induce *hlyE* transcription under pH 5.0, independently of the presence of *phoPQ* (Figure 3A).

In the case of the *hlyE* induction under low Mg^2+^ concentrations, we found that *phoPQ* is essential to increase *hlyE* transcript in an *rpoS*-dependent manner. In *S. enterica* RpoS accumulates when bacteria are grown under low Mg^2+^ conditions [33]. This process requires PhoPQ, which is specifically activated under low Mg^2+^ by increasing the transcription of *iraP*, as previously reported [33,35]. It is possible that PhoPQ is participating in the increase of *hlyE* transcription under low Mg^2+^ via IraP-dependent RpoS stabilization. Nevertheless, in the case of low Mg^2+^ we found that *rpoS* is unable to induce an increased transcription of *hlyE* in a Δ*phoPQ* background. Previous studies reported that low Mg^2+^ concentrations are unable to induce RpoS accumulation in a Δ*phoPQ* background [33], supporting our results.

In this paper we showed that RpoS is the most important activator of *hlyE* expression. According to our results, RpoS sits atop a hierarchical network that integrates diverse environmental and physiological cues. PhoPQ, CRP, and Fis fine-tune these global inputs to precisely control the dosage of HlyE, but also the time in the infection cycle in which this gene is expressed. For HlyE, the amount of protein produced can determine the function. For instance, sublytic concentrations of HlyE affect the Ca^2+^ homeostasis in epithelial cells by induction of slow, intracellular Ca^2+^ oscillations, while increased concentrations act cytolytically [36]. Thus, HlyE belongs to the novel group of pore-forming toxins shown to elicit such biphasic responses [36]. On the other hand, HlyE apparently is participating in different stages of the infective process. Previously, it has been reported that *S.* Typhi *hlyE* mutants are impaired in invasion of human epithelial cells HEp-2 *in vitro*[10]. Consistently, other pore-forming hemolysins play critical roles in invasion of eukaryotic epithelial cells [37-39]. These data show that *hlyE* could participate in the intestinal stage of the *S.* Typhi infection. On the other hand, HlyE is capable to reduce or control bacterial growth, as *hlyE* deletion enhanced *S.* Typhi survival within macrophages without affecting cytotoxicity [11], suggesting that this cytolysin could also be participating in the systemic dissemination of bacteria inside macrophages. In addition, *hlyE* increase their expression when bacteria were cultured under high osmolarity, a condition normally associated to the gut [14,40], but also when bacteria were cultured under low Mg^2+^ concentrations or low pH (Figure 3), conditions found inside the macrophages [41,42]. RpoS, PhoP, CRP and Fis may be important for the proper *hlyE* expression inside macrophages, since the RpoS and PhoP respective regulons are activated inside these cells; and both *crp* and *fis* expression is repressed in the same environment [41,43]. Furthermore, *hlyE* induction by high osmolarity is RpoS dependent [14], reinforcing the fact that this sigma factor is an integrator of different signaling pathways. All this evidence together strongly suggests that HlyE is not circumscribed to a single stage of the infective process, as previously proposed [14]. This work show that *hlyE* presents a complex regulation network involving different environmental signals and global regulators, presumably because this gene is actually participating in different stages of the *S.* Typhi infections process.

## Conclusions

We studied the *hlyE* regulation in *S.* Typhi using genetic approaches. All our results together show that, at least under the tested conditions, RpoS is the most important up-regulator in the *hlyE* regulatory network. RpoS integrates multiple environmental signals and global regulators, including CRP, Fis, and PhoP signaling pathways, to control *S.* Typhi *hlyE* expression.

## Methods

### Bacterial strains, media and culture conditions

A clinical isolate of *S.* Typhi, named STH2370, was obtained from the Infectious Diseases Hospital Lucio Córdova in Santiago, Chile. *S.* Typhi STH2370 strain was grown to an optical density at 600 nm (OD_600_) of 1.4, and frozen as a primary stock. *S.* Typhi STH2370 and derivative strains were grown routinely in liquid culture in Luria-Bertani (LB) broth (Bacto tryptone, 10 g/liter; bacto yeast extract, 5 g/liter; NaCl, 5 g/liter) at 37°C, with aeration provided by shaking. When required, medium was supplemented with chloramphenicol (20 μg/ml), ampicillin (100 μg/ml), gentamicin (20 μg/ml) or glucose (0.4% w/v). LB medium was solidified by the addition of agar (15 g/l). The culture medium was modified in order to simulate the environmental conditions during *S.* Typhi infection process. To determine the effect of glucose in gene expression, bacteria were grown in LB buffered at pH 7.0 to an OD_600_ = 0.35, washed three times with PBS prior to be resuspended with LB buffered at pH 7.0 (control) or LB buffered at pH 7.0 + 0.4% w/v glucose. To determine the effect of low pH in gene expression, bacteria were grown in LB buffered at pH 7.0 to an OD_600_ = 0.35, washed three times with PBS prior to be resuspended with LB buffered at pH 7.0 (control) or LB buffered at pH 5.0 with buffer citrate. To determine the effect of low Mg^2+^ in gene expression, bacteria were grown in LB buffered at pH 7.0 to an OD_600_ = 0.35, washed three times with PBS prior to be resuspended with M9 10 mM Mg^2+^(control) or M9 10 μM Mg^2+^. In all the cases, bacteria were incubated 1 h at 37°C with shaking prior to isolate total RNA or total proteins of each strain. It is important to underline that all the strain tested are in logarithmic phase at OD_600_ = 0.35 when cultured in LB buffered at pH 7.0.

### Construction of *S.* Typhi mutant strains

Mutant strains with substitution of the *hlyE*, *rpoS*, *crp*, *phoPQ* or *fis* genes by resistance cassettes (*cat*: resistance to chloramphenicol) or FRT scar were constructed using the Red/Swap method [44]. PCR primers with 60 bases long overlapping the internal regions of the genes were synthesized with 40 bases at the 5’ ends corresponding to the regions flanking the desired substitutions (Table 1). *S.* Typhi *hlyE*-3xFLAG and *S.* Typhi *rpoS*-3xFLAG mutants were constructed using the primers listed in Table 1 as previously described [22]. All the double mutants were constructed by electrotransformation with gDNA from single mutants as described [23]. The presence of each substitution was confirmed by PCR using primers complementary to the DNA genome flanking the sites of substitution.

**Table 1:** Primers used in this study

**Primers used for the red/swap technique [**[44]**]**	
*crp* (H1 + P1)	TTCTTGTCTCATTGCCACATTCATAAGTACCCGTCAAAGAtgtaggctggagctgcttcg
*crp* (H2 + P2)	GGCGAGGTTACCTACTTTTTCAGAGGTGACTTGTAAGCGAcatatgaatatcctccttag
*fis* (H1 + P1)	AAATTCTGACGTACTGACCGTTTCTACCGTTAACTCTCAGtgtaggctggagctgcttcg
*fis* (H2 + P2)	ACGCAGCGTACCACGGTTGATGCCCATCATCAGAGCAGCAcatatgaatatcctccttag
*rpoS* (H1 + P1)	AATACGCTGAAAGTTCATGATTTAAATGAAGACGCGGGtgtaggctggagctgcttcg
*rpoS* (H2 + P2)	GCGCAGGTATACGTTCAGCTCTTTAACAATGTGAATcatatgaatatcctccttag
*phoP* (H1 + P1)	CCAGGATTCAGGTCACCAGGTCGATGCCGCAGAAGATGtgtaggctggagctgcttcg
*phoQ* (H2 + P2)	TTCCTCTTTCTGTGTGGGATGCTGTCGGCCAAAAACGACCcatatgaatatcctccttag
Primers for epitope tagging (3xFLAG) [22]	
*rpoS*-3xFLAG	GCAGACGCAGGGGCTGAATATCGAAGCGCTGTTCCGCGAG*gactacaaagaccatgacgg*
*rpoS*-kan	TCTGGACGGTATATCAGTGTCAGCATTGTCTGTATACCTG*catatgaatatcctccttag*
*hlyE*-3xFLAG	AAGACACGGTAAGAAGACGCTTTTCGAGGTTCCTGACGTC*gactacaaagaccatgacgg*
*hlyE*-kan	GAATGCGGAAATCACCCTCGACTACCAGCTTAACGCCTGAC*catatgaatatcctccttag*
Primers used for cloning	
CRP-C	**CTGCAG**GCTGGCCTATCAATAAACCA
CRP-N	**CTGCAG**AAGCGAGACACCAGGAGACA
RPOS-C	**CTGCAG**CCGATGATTTGTCCACGCTG
RPOS-N	**CTGCAG**TGCCCCGTATAGCCTGAATG
PHOPQ-C	CGTTCAAGAAAGTCGGGCCA
PHOPQ-N	GCCTTAAAGCCATGACGCCG
FIS-C	TT**CTGCAG**GCGCCTTTTTAAACAAGCAG
FIS-N	TT**CTGCAG**GACCAGTTTCGGCGCACATT
Primers for mRNA detection by RT-PCR	
RT-HLYE-C2	CGCTTCATTCAGTTTCTTGA
RT-HLYE-N	AAGTTTTGCTTATGGACAGC
RT-RPOS-C	TTCGGAATCACCGCCCAGCG
RT-RPOS-N	TCGCCGTCGCATGATTGAGAG
RT-FIS-C	CATCATCAGAGCAGCACGGG
RT-FIS-N	TCAGGATCAGGTAACCCAAA
16SFW	GTAGAATTCCAGGTGTAGCG
16SRV	TTATCACTGGCAGTCTCCTT

Lowercase: Annealing site with pKD3 [44].

Italics: Annealing site with pSUB11 [22].

Bold letters: PstI restriction site.

### Plasmid construction

PCR amplifications for plasmid construction were performed using an Eppendorf thermal cycler and Taq (Fermentas) DNA polymerase. Reaction mixtures contained 1x PCR buffer, 1.5 mM MgCl_2_, each dNTP (200 mM), primers (1 mM), 100 ng of template DNA, and 2 U of Taq DNA polymerase. Standard conditions for amplification were 30 cycles at 96°C for 40 s, 60°C for 45 s, and 72°C for 2 min, followed by a final extension step at 72°C for 10 min. Template *S.* Typhi STH2370 chromosomal DNA was prepared as described [45]. For complementing *S.* Typhi Δ*rpoS* or *S.* Typhi Δ*phoPQ*, we constructed the pBRPOS and pBPHOPQ plasmids by cloning the PCR product generated with RPOS-C + RPOS-NP or PHOPQ-C + PHOPQ-NP, respectively, into pBBR5. For complementing *S.* Typhi Δ*crp* or *S.* Typhi Δ*fis*, we constructed the pTCRP and pTFIS plasmids by cloning the PCR product generated with CRP-C + CRP-NP or FIS-C + FIS-NP, respectively, into pCR TOPO 2.1 TA (Invitrogen) according to the manufacturer’s instructions. In all the cases, the cloned amplicons contained the ORF and the corresponding promoter region.

### RNA isolation, reverse transcription and real-time quantitative PCR (RT-PCR)

Total mRNA from the strains grown under the tested conditions was extracted using TRIzol reagents (Invitrogen) as described by the manufacturer. RNA was precipitated with isopropanol for 10 min at room temperature, washed with ice cold 70% v/v ethanol and resuspended in DEPC-treated water prior to treatment with DNase I to remove any trace of DNA. Purity of extracted RNA was determined by spectrometry. Reverse transcription was performed on 2 μg of DNase-treated RNA using Superscript II RT (Invitrogen) at 50°C for 50 min followed by 70°C for 10 min in 20 μl with 2.5 mM of the corresponding reverse primer (RT-HLYE-C, RT-RPOS-C and RT-FIS-C for *hlyE, rpoS* and *fis* mRNA detection, respectively). The 16sRV reverse primer for 16 s mRNA by quantitative detection was used under each condition to normalize against a reference transcript. Relative quantification of each mRNA was performed using Brilliant II SYBR Green QPCR Master Reagent Kit and the Mx3000P detection system (Stratagene). The reaction mixture was carried out in a final volume of 20 μl containing 1 μl of diluted cDNA (1:1000), 0.24 μl of each primer (120 nM) (RT-HLYE-C + RT-HLYE-N for *hlyE* mRNA detection, RT-RPOS-C + RT-RPOS-N for *rpoS* mRNA detection, RT-FIS-C + RT-FIS-N for *fis* mRNA detection and 16sFW + 16sRV for 16 s mRNA detection), 10 μl of 10X Master Mix, 0.14 μl of diluted ROX (1:200) and 8.38 μl of nuclease-free H_2_O. The reaction was performed under the following conditions: 10 min at 95°C followed by 40 cycles of 30 s at 95°C, 30 s at 53°C and 45 s at 72°C. Finally a melting cycle from 53 to 95°C was performed to check for amplification specificity. Amplification efficiency was calculated from a standard curve constructed by amplifying serial dilutions of RT-PCR products for each gene. These values were used to obtain the fold change in expression for the gene of interest normalized with 16 s levels as previously described [46]. Experiments were performed in three biological and technical replicates for each culture condition. When appropriate, the statistical significance of differences in the relative expression data was determined using the one way ANOVA test.

### Immunodetection analysis

3xFLAG fusion proteins were immunodetected by the use of anti-FLAG M2 mAbs from Sigma. Strains carrying the epitope-tagged gene were grown under the conditions described above and washed twice with 100 mM Tris–HCl pH 8.0. Bacterial pellets were resuspended with 100 mM Tris–HCl and sonicated 60 s on ice. Then, the samples were mixed with 1 volume of Laemmli buffer [47]. Suspensions were incubated at 98°C for 5 min. The resulting lysates were quickly centrifuged to remove cell debris and used, straight or suitably diluted, for SDS-PAGE. Twenty μg of bacterial proteins, previously quantified following the Bradford method [48], were resolved by SDS-PAGE and then transferred to poly (vinylidenedifluoride) membranes and probed with mAbs (1:1.000) and horseradish peroxidase-conjugated goat antimouse IgG [1:5.000 (Sigma)]. Detection was performed by enhanced chemioluminescence (ECL, Amersham Pharmacia).

### Hemolytic activity quantification

*S.* Typhi and derivative strains were grown under the tested conditions, the OD_600_ was recorded, and the cultures were chilled to 4°C. Samples of each strain were washed twice with PBS, resuspended in 1 ml of PBS and then sonicated for 60 s on ice. This suspension was gently mixed with 1 volume of sheep erythrocytes (10^9^ cells/ml) previously washed twice with PBS, and incubated 30 min at 37°C without shaking. To measure the hemolytic activity, samples were cleared by centrifugation and the OD_545_ was determined. Instead of the bacterial suspension, PBS or 5% sodium deoxycholate (DC) were used as reference hemolysis values for 0 and 100%, respectively. The arbitrary hemolytic units were calculated using the formula: (OD_545_Sample – OD_545_PBS)/(OD_545_DC – OD_545_PBS).

## Competing interests

The authors declare that they have no competing interests.

## Authors’ contributions

MRJ designed the studies, performed the experiments and contributed to write the manuscript. LMR performed the experiments of Figure 1. NAV performed the hemolysis assays. AAH participated in discussing the results and revising the manuscript. GCM participated in the revision of the manuscript. JAF designed the studies and wrote the paper. All authors read and approved the final manuscript.
